# Pituitary Stalk Interruption Syndrome in Chinese People: Clinical Characteristic Analysis of 55 Cases

**DOI:** 10.1371/journal.pone.0053579

**Published:** 2013-01-14

**Authors:** Qinghua Guo, Yan Yang, Yiming Mu, Jvming Lu, Changyu Pan, Jingtao Dou, Zhaohui Lv, Jianming Ba, Baoan Wang, Xiaoman Zou, Lijuan Yang, Jinzhi Ouyang, Guoqing Yang, Xianling Wang, Jin Du, Weijun Gu, Nan Jin, Kang Chen, Li Zang, Bradley J. Erickson

**Affiliations:** 1 The Division of Endocrinology, Chinese PLA General Hospital, Beijing, China; 2 Radiology, Mayo Medical School, Mayo Clinic, Rochester, Minnesota, United States of America; University of Cordoba, Spain

## Abstract

**Objective:**

Pituitary stalk interruption syndrome (PSIS) is characterized by the absence of pituitary stalk, pituitary hypoplasia, and ectopic posterior pituitary. Due to the rarity of PSIS, clinical data are limited, especially in Chinese people. Herein, we analyzed the clinical characteristics of patients diagnosed with PSIS from our center over 10 years.

**Patients and Methods:**

We retrospectively analyzed the clinical manifestations and laboratory and MRI findings in 55 patients with PSIS.

**Results:**

Of the 55 patients with PSIS, 48 (87.3%) were male. The average age was 19.7±6.7 years and there was no familial case. A history of breech delivery was documented in 40 of 45 patients (88.9%) and 19 of 55 patients (34.5%) had a history of dystocia. Short stature was found in 47 of 55 patients (85.5%) and bone age delayed 7.26±5.37 years. Secondary sex characteristics were poor or undeveloped in most patients. The prevalence of deficiencies in growth hormone, gonadotropins, corticotropin, and thyrotropin were 100%, 95.8%, 81.8%, 76.3%, respectively. Hyperprolactinemia was found in 36.4% of patients. Three or more pituitary hormone deficiencies were found in 92.7% of the patients. All patients had normal posterior pituitary function and absent pituitary stalk on imaging. The average height of anterior pituitary was 28 mm, documented anterior pituitary hypoplasia. Midline abnormalities were presented in 9.1% of patients.

**Conclusions:**

The clinical features of our Chinese PSIS patients seem to be different from other reported patients in regarding to the higher degree of hypopituitarism and lower prevalence of midline defects. In addition, our patients were older at the time of case detection and the bone age was markedly delayed. We also had no cases of familial PSIS.

## Introduction

Pituitary stalk interruption syndrome (PSIS) is characterized by an absent pituitary stalk on MRI, hypoplasia of the anterior pituitary gland, and ectopic posterior pituitary [1], [2]. It has been shown that patients with PSIS form a distinct anatomical and endocrinological syndrome–presenting with signs and symptoms of either isolated growth hormone deficiency or multiple anterior pituitary hormone deficiency. Posterior pituitary function is normal in these patients [3]–[5].

Since PSIS is a relatively newly recognized disorder with the introduction of MRI, the exact prevalence is uncertain. Most population studies related to PSIS are from the western hemisphere [6]–[9]. There is no large study of the clinical features of PSIS in Asian people, although there are some individual case reports [10]–[12].

Over the past decade we collected 55 cases of Chinese patients with PSIS. In the present study, we analyzed the clinical presentations and laboratory and imaging data. This report represents the largest group of Chinese or Asian PSIS patients studied to date.

## Results

### 1. General Data

The 55 patients with PSIS came from different parts of China, accounting for 21.9% of hospitalized hypopituitary patients diagnosed in our hospital during the same period. The ratio of male to female was 6.9∶1. The average age was 19.7±6.7 years (range, 9 to 43 years). There was no documentation of consanguineous parents and familial history in any of the cases. The fetal position was not clear for 7 patients, and 3 patients was born by Caesarean; 5 patients presented with head presentation (5/45, 11.1%) and 40 patients experienced breech delivery or footling delivery (40/45, 88.9%), among them 19 patients had the history of dystocia (19/55, 34.5%). Sex chromosome findings matched gender phenotype.

### 2. Clinical Features

The clinical manifestations are summarized in Table 1. The most common presentation was growth retardation (49/55, 89.1%). Other presentations included absent pubertal development (5/55, 9.1%) and blurred vision (1/55, 1.8%). Most of the 55 patients presented with slow growth and development, with annual height increases <3 cm and absent grow spurt. Their mean height (±SD) was 144.0±15.6 cm (range, 109 to 173 cm). The calculated mean (±SD) SDS for height based on chronological age was −3.76±2.12 (−9.3∼0.05). There were 47 patients (47/55, 85.5%) with stature more than 2 standard deviations below the mean. The heights of 25% PSIS patients were within the normal range, and 32.7% (18/55) were taller than 155 cm and 12.7% (7/55) were taller than 170 cm. The heights of upper body and lower body segments were 70.6±7.2 cm and 74.9±8.3 cm, respectively. The mean (±SD) ratio of upper body segment to lower body segment was 0.94±0.06. The mean (±SD) arm span was 142.9±15.6 cm, and the mean (±SD) ratio of arm span to height was 0.97±0.14.

**Table 1:** Clinical characteristics of 55 patients with PSIS.

Case	Sex	Age (y)	Main complain	Perinatal events	Height (cm)	SDS	Weight (kg)	Upper/lower segment	Armspan/Height	Penile length (cm)	Testicular volume (left/right) or Breast	Bone age
1	M	20	Retardation	Breech	120	−8.64	26	0.97	0.97	3	2/2	12
2	M	18	Retardation	Breech	165	−1.26	53	0.88	0.96	6	1.5/1	13.5
3	M	16	Retardation	Breech	159	−2.03	32	0.94	0.95	3	2/2	13.5
4	M	18	No puberty	Breech	154	−3.07	73	0.98	1.05	3	2/2	13
5	F	17	No puberty	Head	159	−0.24	28	1.04	0.94	ND	Tanner I	13
6	M	22	Retardation	Breech	155	−2.90	43	0.82	1.01	2.5	1/0.7	13
7	M	27	Retardation	ND	141	−5.20	43	0.88	0.99	3.5	1.5/1.5	15
8	M	15	Retardation	Breech	136	−5.20	38	0.92	0.96	4	2/2	11
9	M	18	Retardation	Breech	149	−3.89	44	0.86	0.99	6	4/4	13
10	M	19	Retardation	ND	135	−6.18	42	0.99	1.01	3	1/1	12
11	M	21	Retardation	Breech/dystocia	168	−0.77	59	0.87	0.97	5	3/3	13
12	M	18	Retardation	Breech/dystocia	134	−6.34	33.5	0.97	1.01	3.5	1/1	11
13	M	20	Retardation	Breech/dystocia	157	−2.57	45	0.86	1.01	4	1/1	14.5
14	M	17	Retardation	Caesarean	141	−5.22	37	0.99	1.00	3	1/1	13
15	F	16	Retardation	Head	133	−5.02	34	1.03	0.97	ND	Tanner?-IV	16
16	M	10	No puberty	Caesarean	122	−2.94	26.5	0.98	0.97	1.5	Cryptorchidism	5
17	M	23	Retardation	Breech	155	−2.82	54	0.87	1.00	2.5	1/2	15
18	M	27	Retardation	Breech/dystocia	158	−2.41	51	1.16	0.95	4	2/1	13
19	F	40	Retardation	Breech/dystocia	159	−0.30	67	0.88	1.04	ND	Tanner?-IV	16
20	M	17	Retardation	ND	142	−5.05	40	0.92	0.99	3	Cryptorchidism	11
21	M	21	Retardation	Head	160	−2.08	63	ND	ND	2	1/2	14
22	M	21	Retardation	Breech/dystocia	155	−2.90	47	0.90	1.01	3.5	2/2	13
23	M	18	Retardation	Breech	162	−1.75	56	0.98	0.00	3	1/1	14
24	M	22	Retardation	ND	146	−4.38	45.5	0.96	0.98	5.5	1/1	13
25	F	43	Retardation	Breech	142	−3.44	37	0.89	1.03	ND	Tanner I	14
26	M	35	Retardation	Breech	116	−9.30	28	0.97	0.97	5	1.5/1.5	14
27	M	17	Retardation	Breech/dystocia	127.5	−7.47	45	1.04	1.01	3	1.5/1.5	13
28	M	12	Retardation	ND	127	−3.41	43	0.95	1.13	1	2/1.5	8
29	M	23	Retardation	Breech	163	−1.59	47	0.90	1.02	2.5	2.5/2.5	14
30	M	23	Retardation	Breech/dystocia	142	−5.03	44	1.00	0.99	2.5	1.7/1.6	13
31	M	19	No puberty	Breech	151	−3.56	53	1.01	0.95	4.5	Cryptorchidism	13
32	M	10	Retardation	Breech/dystocia	109	−5.33	20	1.00	1.01	2.5	1/1	5
33	M	10	Retardation	Breech	125	−2.79	35	0.92	1.15	3.5	1/1	8
34	M	19	Retardation	Breech	158	−2.41	57	0.90	0.98	3.5	1/1	13
35	M	26	Retardation	Breech/dystocia	153	−3.23	71	0.96	1.01	3.5	10/9	15
36	M	18	Retardation	Breech/dystocia	150	−3.72	25	0.97	0.99	5	3/3	13
37	M	25	Retardation	Breech	152	−3.39	48	0.95	0.99	2	2.5/2.5	14
38	M	25	No puberty	Breech	162.5	−1.67	56	0.88	0.98	4	2.5/2	14
39	M	13	Retardation	Breech/dystocia	133	−3.44	31	0.87	0.98	5	2/2	9
40	M	18	Retardation	Breech/dystocia	151	−3.56	66.5	0.99	0.97	2	1/1	13.5
41	M	20	Retardation	Breech	132	−6.67	30.6	1.00	0.91	2	3/3	15
42	M	11	Retardation	Breech	131	−2.17	40	0.96	0.98	2.5	3/3	9
43	F	16	Retardation	Breech/dystocia	113.5	−8.67	21	0.97	0.98	ND	TannerI	6
44	M	15	Retardation	Head	121	−7.51	21.1	0.95	0.96	2.5	1/1	6
45	F	9	Blur vision	ND	114	−3.69	19	ND	ND	ND	TannerI	5
46	M	20	Retardation	Breech/dystocia	142	−5.03	38	0.97	1.00	2	1/1	15
47	M	14	Retardation	Breech/dystocia	139	−3.74	30	1.01	0.97	7	7/6	11
48	M	23	No puberty	Head	173	0.05	73	1.07	1.00	5	4/4	18
49	F	27	Retardation	Breech	145	−4.54	37.9	0.91	0.99	ND	TannerI	12
50	M	12	Retardation	Caesarean	136	−2.18	32.5	0.86	0.97	3	3/3	12
51	M	25	Retardation	Breech/dystocia	159	−2.25	70.8	0.89	1.03	2.5	1/2	14
52	M	21	Retardation	ND	143	−4.87	38	0.86	0.99	3	1/1	14
53	M	14	Retardation	Breech	143	−3.18	42.4	0.83	0.98	3	3/3	12.5
54	M	20	Retardation	Breech/dystocia	159	−2.25	50	0.94	1.02	4.5	1/1	14
55	M	11	Retardation	Breech/dystocia	126	−2.92	25	0.94	0.98	3.0	2/2	8

ND: not documented. F: female. M: male. SDS: standard deviation scores. PSIS: Pituitary stalk interruption syndrome.

Only 2 patients in this group of patients had even partial sex development and most patients were lack of sexual development. There were 5 cases that were not identified as having PSIS until they presented to the clinic for absent development of secondary sexual characteristics after pubertal age. The male patients exhibited no pubertal development, absent pubic and axillary hair, lack of adult male larynx development. The mean (±SD) testicular volumes were 2.21±1.94 ml (right) and 2.27±2.04 ml (left), respectively. The mean (±SD) length of flaccid penis was 3.48±1.29 cm. The prevalence of microphallus was 23.4%. Three patients had cryptorchidism, accounting for 6.3% of the male patients. Most of the female patients also exhibited poor secondary sexual characteristic development–including: absent pubic or axillary hair, hypoplasia of mammary glands, infantile uterus or primordial uterus, ovarian dysgenesis, non-development of labia majora and labia minora. However, a few patients had axillary hair and breasts were in Stage B3 (Tanner Staging 5 system), and pubic hair was in Stage P3 (Tanner Staging 5 system); labia majora and labia minora showed pigmentation, but they had an infantile uterus. All female patients had primary amenorrhea.

In addition to relatively short stature and delayed or poor sexual development compared to normal peers, some patients were troubled by fatigue and hypodynamia and were more susceptible to upper respiratory infections. However, most of the patients had intelligence within the normal range, although their school records tended to reflect slightly lower performance.

No patient complained of thirst, polydipsia or polyuria. The mean (±SD) fasting plasm glucose was 4.52±0.42 mmol/L and the mean (±SD) cholesterol was 3.81±0.45 mmol/L.

### 3. Laboratory Evaluation of Hormonal Status

#### 3.1 Anterior pituitary function

Anterior pituitary function was evaluated with basal and dynamic testing as described above. The ITT and pyridostigmine bromide stimulating GH test revealed that 100% of the patients had GH deficiency; median basal GH was 0.05 ug/L (range, 0.05–1.10; interquartile range [IQR]: 0.05–0.07). With the stimulation tests, the GH secretion curves were low and flat, of which the peak median GH values were 0.05 ug/L (range, 0.05–1.30; IQR: 0.05–0.10) and 0.05 ug/L (range, 0.05–4.20; IQR: 0.05–0.10) for ITT and pyridostigmine bromide stimulation tests, respectively; All of the patients were diagnosed with complete GH deficiency.

The Gonadorelin stimulation LH test indicated that the patients had low and flat LH secretion curves, except for Patients No.15, 30, 47 and 51. The basic median LH level was 0.07 mIU/ml (range, 0.01–0.59; IQR: 0.07–0.10), and the peak median value of LH was 0.23 mIU/ml (range, 0.07–2.60; IQR: 0.09–0.50); the median testosterone level in male patients was 0.36 ng/ml (range, 0.06–2.86; IQR: 0.35–0.70), and the median estradiol level in female patients was 36.7 pg/ml (range, 20.0–102.4; IQR: 36.7–51.4). The basal LH levels for Patients No.15, 30, 47 and 51 were 7.45 mIU/ml, 0.59 mIU/ml, 1.65 mIU/ml and 0.83 mIU/ml and the corresponding peak values after stimulation were 71.62 mIU/ml, 4.25 mIU/ml, 7.61 mIU/ml and 5.13 mIU/ml, respectively. Gonadotrophin deficiency occurred to 46 patients (46/55, 83.6%). When the status of the gonadotrophic axis was evaluated in boys older than 14 years and in girls older than 13 years, the prevalence of gonadotrophin deficiency reached to 95.8% (46/48).

The mean basal ACTH level at 8 am was 3.77±1.88 pmol/L and median cortisol level at 8 am was 51.70 ng/ml (range 25.70–355.70; IQR: 29.32–161.50). ACTH levels with the ITT gave low and flat curves, the mean peak value after stimulation was 4.31±2.47 pmol/L and the ratio of peak ACTH to basal ACTH was 1 (range 0–3; IQR: 1–1). Secondary adrenal insufficiency was diagnosed in 81.8% (45/55) of the patients based on the 8 am cortisol levels and ACTH responses to ITT.

Among the 55 patients, 42 patients (76.3%) were diagnosed with secondary hypothyroidism based on FT4 levels <10.4 pmol/L (the mean (±SD). TSH level in these patients was 5.11±4.10 uIU/ml and mean (±SD) FT4 level was 8.01±1.55 pmol/L). The FT4 levels in the remaining 13 patients were also near the lower limit of reference range, with mean (±SD) of 11.73±1.38 pmol/L (mean (±SD) TSH was 3.67±1.62 uIU/ml). In 15 patients, the TSH levels were >5.5 uIU/ml with a mean (±SD) of 8.86±4.36 uIU/ml (range, 5.65 uIU/ml to 21.93 uIU/ml). The FT4 levels were comparable between the higher TSH group and normal TSH group (*P*>0.05). Among the TSH higher group, thyroid ultrasound examination, and thyroid autoimmune antibodies were negative.

Twenty patients had hyperprolactinemia, accounting for 36.4% of PSIS patients, while no patient with hypoprolactinemia was found.

The prevalences of abnormalities in the hypothalamus-pituitary system among the PSIS patients including growth hormone deficiency, hypogonadism, hypoadrenalism, hypothyroidism and hyperprolactinemia were 100%, 95.8%, 81.8%, 76.3% and 36.4%, respectively. The number of patients with one, two, three, four or five kinds of hormonal abnormalities were 1(1.8%), 3(5.5%), 13(23.6%), 21(38.2%) and 17(30.9%), respectively. Therefore, 51 patients had more than 3 hypothalamic-pituitary hormonal abnormalities, accounting for 92.7% (51/55) of this group of PSIS patients.

#### 3.2 Comparison between different hormone statuses

The FT_4_, cortisol (F8 am) and LH peak in hypothyroidism group were much lower than that in non-hypothyroidism group while SDS of height and heights were higher (*P*<0.05); The ratio of upper body segment to lower body segment, bone age, FT_4_, basal LH and LH peak in hypoadrenalism group were lower than that in non-hypoadrenalism group, and the height and SDS of height were higher (*P*<0.05); In hypogonadism group, the age, ratio of male to female, breech delivery prevalence, height and bone age were much higher than that in non-hypogonadism group while basal GH, GH peak, cortisol (F8 am), testosterone (T), basal LH and LH peak were lower (*P*<0.05); The ratio of male to female and breech delivery prevalence were higher in hyperprolactinemia group while bone age, E_2_ and prolactin (PRL) were lower than that in normal prolactin group (*P*<0.05). There was no differences in terms of age, basal GH, peak GH, basal LH, peak LH, T, E_2_, PRL, basal ACTH, peak ACTH, 8 am cortisol, FT_4_ and TSH between the patients of short stature and normal stature patients (Table 2).

**Table 2:** Comparison between different endocrine subgroups.

	Hypothyroidism		Hypocortisolism		Hypogonadism		Hyperprolactinemia	
	yes	no	yes	no	Yes	no	yes	no
N.	42	13	45	10	46	9	20	35
**Percent (%)**	76.36	23.63	81.81	22.22	83.63	16.36	36.36	63.63
**Age(year)**	19.64±6.92	19.23±6.31	18.82±5.61	22.80±10.15	21.13±6.15	11.44±2.24*	18.25±5.96	20.53±7.06
**M/F**	37/5	11/2	41/4	7/3	41/5	7/2*	19/1	28/6*
**Breech prevalence (%)**	31/35(88.57)	9/10(90.00)	32/36(88.88)	8/9(88.88)	35/40(87.50)	5/6(83.33)*	17/18(94.44)	23/27(85.18)*
**Height(cm)**	145.77±15.41	137.00±14.9*8	145.62±15.58	135.05±13.32*	147.34±13.98	125.11±9.27*	140.42±18.01	145.85±14.13
**-SDS Height**	−3.36±1.89	−5.02±2.32*	−3.44±2.01	−5.09±2.07*	−3.79±2.26	−3.56±1.04	−4.17±2.31	−3.54±1.98
**RUL**	0.94±0.07	0.95±0.07	0.93±0.06	0.98±0.04*	0.94±0.07	0.99±0.04	0.95±0.07	0.94±0.06
**RAH**	0.97±0.16	0.98±0.03	0.97±0.15	0.98±0.03	0.97±0.15	1.02±0.08	0.94±0.22	0.99±0.03
**Bone age(year)**	12.18±3.03	12.62±2.70	12.12±2.90	13.00±3.12*	13.05±2.09	8.33±3.54*	11.10±3.32	12.98±2.52*
**Basal GH(ug/L)**	0.12±0.23	0.14±0.23	0.11±0.22	0.16±0.26	0.07±0.04	0.43±0.48*	0.19±0.29	0.05(0.05–0.05)
**GH peak 1(ug/L)**	0.15±0.26	0.16±0.26	0.14±0.25	0.19±0.29	0.09±0.12	0.47±0.48*	0.22±0.31	0.05(0.05–0.09)
**GH peak 2(ug/L)**	0.14±0.27	0.40±1.14	0.13±0.26	0.49±1.30	0.08±0.10	0.80±1.38	0.36±0.93	0.05(0.05–0.06)
**TSH (uIU/ml)**	5.19±4.07	3.67±1.62	5.04±3.97	3.86±1.66	4.90±3.67	4.47±3.96	4.55±3.14	4.88±4.01
**FT4 (pmol/L)**	8.01±1.55	11.73±1.39*	8.42±1.72	10.96±2.88*	8.66±2.00	10.03±2.84	8.91±2.36	8.89±2.14
**ACTH(pmol/L)**	3.62±1.69	4.27±2.44	3.68±1.94	4.14±1.57	3.75±1.96	3.87±1.45	3.37±1.78	4.02±1.93
**ACTH peak(pmol/L)**	3.96±2.29	5.39±2.82	4.02±2.28	5.64±3.02	4.45±2.57	3.42±1.68	3.97±2.25	4.53±2.66
**F8** **am(nmol/L)**	80.05±69.93	181.51±120.25*	67.25±50.78	269.53±55.67*	85.88±78.71	196.83±114.40*	98.01±95.97	109.63±94.46
**T(ng/ml)**	0.52±0.30	0.75±0.76	0.51±0.29	0.86±0.85	0.51±0.29	0.92±0.89*	0.66±0.63	0.52±0.28
**E_2_(pg/ml)**	42.27±19.08	51.65±20.53	41.79±17.04	55.24±25.48	44.17±18.33	47.31±26.54	37.47±4.82	50.70±22.11*
**LH(mIU/ml)**	0.11±0.13	0.85±2.04	0.10±0.12	1.08±2.28*	0.13±0.16	1.06±2.45*	0.20±0.37	0.07(0.07–0.11)
**LH Peak(mIU/ml)**	0.48±0.85	6.90±19.57*	0.43±0.78	9.03±22.11*	0.62±1.03	9.04±23.59*	0.98±1.86	0.11(0.26–0.68)
**PRL(ug/L)**	18.09±10.71	13.53±5.51	17.63±10.51	14.20±5.85	16.61±9.37	18.90±12.54	27.44±7.08	10.84±4.72*

* P<0.05 compared to control group.

ACTH: adrenal corticotropic hormone. E2: estradiol. F: female. F 8 am: cortisol at 8 am. FT4: free thyroxin. GH: growth hormone. LH: luteinizing hormone. M: male. PRL: Prolactin. RAH: ratio of armspan to height. RUL: ratio of upper body segment to lower body segment. SDS: standard deviation scores. TSH: thyroid-stimulating hormone. T: testosterone.

Height was negatively correlated with basal GH (*P* = 0.022), peak GH1 (*P* = 0.008), peak GH2 (*P* = 0.000), 8 am cortisol (*P* = 0.000) and borderline negatively correlated with FT_4_ (*P* = 0.061). While SDS height was negatively correlated with basal E_2_ (*P* = 0.030), peak PRL (*P* = 0.004), peak ACTH (*P* = 0.021), 8 am cortisol (*P* = 0.001), FT_4_ (*P* = 0.025) and borderline negatively correlated with T (*P* = 0.057).

#### 3.3 Function of posterior pituitary

Before and after the hormone replacement therapy for anterior pituitary hormone deficiencies, signs and symptoms of diabetes insipidus were absent. The 24-hour water intake and urine amount were less than 2000 ml, and the osmotic pressures of blood and urine and the specific gravity of urine were all in respective reference ranges.

### 4. Imaging Evaluation

**Figure 1:**
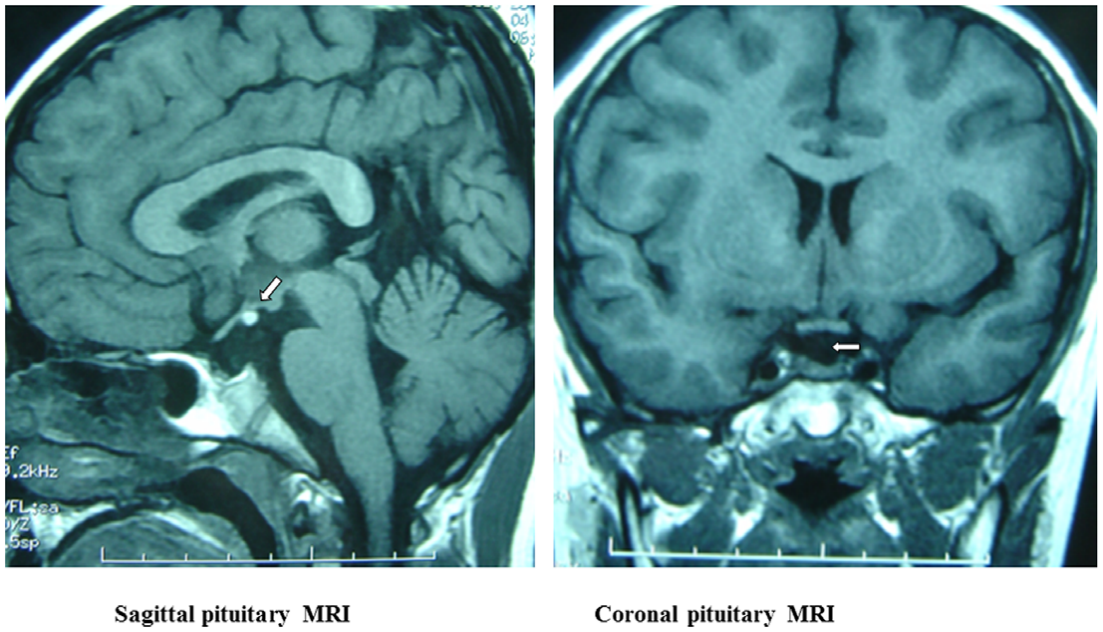
The sagittal (left) and coronal (right) pituitary MRI. The sagittal image shows the ectopic pituitary (arrow), which is located at the floor of the third ventricle. The coronal image shows absence of a normal pituitary stalk. Also, seen on both images, there is a small anterior pituitary gland.

Pituitary MRI examination revealed dysplasia of anterior pituitary with a mean (±SD) height of 28±9 mm (Table 1); the pituitary stalk was absent, and the ectopic posterior pituitary was located near the optic chiasm and hypothalamus in all of these patients (Fig. 1). Partial empty sella turcica could be observed in 9 patients. Five patients had a combination of midline abnormalities (9.1%), which included optic atrophy, Chiari-I malformation, partial absence of corpus callosum, spina bifida or congenital scaphoid fossa leak. There were no other records of developmental defects of structures or organs along the midline of central nervous system.

The mean (±SD) bone age was 12.28±2.93 years (range, 5 to 16 years), which was lower than the actual age by a mean (±SD) of 7.26±5.37 years.

## Discussion

PSIS was first reported by Fujisawa et al. in 1987 [1]. This syndrome includes a series of clinical findings due to the pituitary stalk absence combined with ectopic posterior pituitary; thus the hormone secreted from hypothalamus could not be transmitted to anterior pituitary through pituitary stalk [15]. Patients with PSIS have various degrees of anterior pituitary hormone deficiency.

The exact prevalence of PSIS is uncertain, but a recently published report indicated the estimated incidence was approximately 0.5/1000,000 births [16]. In China, Liu Ying et al. [11] reported the first case of PSIS in 2004. Thereafter, there were a few individual cases reported [10], [17], [18], of which most were on imaging analysis. In 2008, we firstly analyzed 5 cases of PSIS and published in Chinese Journal of Endocrinology and Metabolism. Later, 13 cases of PSIS were reported [19]. Due to the limited number of reported cases, the clinical features of these patients were variable and there was no general description of Chinese people with PSIS. Even in the reports of other than Asian people, the clinical manifestations of PSIS are complex and diverse.

In our study, Chinese patients with PSIS presented with sometimes different clinical characteristics compared to previous reports. We found a male predominance; the ratio of male to female was 6.9∶1 which is higher than 2.3∶1 reported in the literature [7]. The age for our patients at diagnosis was 19.7 years which is much older than the age of 9.6 years reported by Reynaud R [7], 3.6 years by Gascoin-Lachambre G [9], and 4.0 years by Pinto G [13]. Most (88.9%) of our 55 patients with PSIS presented with breech delivery and 34.5% had a history of dystocia. Chen S. et al. [20] reported similar findings. To date, the underlying mechanisms of PSIS remained to be identified. As patients with PSIS reported have relatively high incidence rates of breech delivery, dystocia and neonatal asphyxia, some investigators think that the abnormal factors during perinatal period and trauma are related to the occurrence of PSIS. Breech delivery leads to obvious deformation of head, which may result in injury or breaking of pituitary stalk; hypoxemia or hypoperfusion due to anoxia after birth may also lead to injury of pituitary stalk and pituitary. According to the literature, 70%∼80% of the dystocia due to breech delivery may lead to injury of pituitary [21], [22]; abnormal fetal position or dystocia happened to 40%∼50% of the PSIS patients. However, it was reported that 54% of the PSIS patients had normal delivery, and in our study, 11.1% of the patients had normal fetal position and 55.5% of them had normal delivery without dystocia history. Therefore, the above-mentioned hypothesis was questioned. Some investigators suggested that congenital dysplasia of hypothalamus and pituitary might result in abnormal activity of fetus in womb, leading to malposition of fetus; therefore, PSIS should be the cause other than result of breech delivery [23]; the possible gene defect of patients might result in dysplasia of hypothalamus and pituitary, leading to abnormal activity of fetus in womb, and thus the proportion of breech delivery increased significantly.

Eighty-nine percent of the 55 patients presented for growth retardation and average heights were 144 cm. About 85% of PSIS patients had short stature. The average bone age was 12.28 years with a mean of 7.26 years younger than actual age. The delay of the bone age is much obvious than the 1.4 years in the literature [9]. In our series, the ITT and pyridostigmine bromide stimulating GH test revealed that 100% of the patients had complete GH deficiency and the GH secretion curves were low and flat. Other studies have also shown the persistence of severe GH deficiency in patients with PSIS. Tauber et al. [8] reported 77% of complete GH deficiency. Arrigo et al. [24] reported a mean peak GH after stimulation of 1.6±1.7 ng/ml in GH deficiency with PSIS. Coutant, et al. [25] observed 15/15 (100%) complete GH deficiency in a group of PSIS. That contrasted with idiopathic GH deficiencies with normal MRI [26] and means PSIS patients suffered from more severe GH deficiency than isolated GH deficiency (IGHD), which presented with complete or partial GH deficiency with short statures of 130–140 cm if not intervened.

Despite the uniformity of GH deficiency, patients had variable heights. The heights of 25% of our patient PSIS patients were within normal range, and 32.7% were taller than 155 cm and 12.7% were taller than 170 cm. The reason for this interesting phenomenon remains to be clarified. The difference between IGHD and PSIS was the latter suffered from multiple pituitary hormone deficiency as well as growth hormone. The height and growth of bone are influenced by many factors including growth hormone, thyroxin, cortisol, E2 and T. So, one possible explanation for the height of PSIS patients may be the complication of multiple hormones deficiencies. We further compared the heights and hormone levels in different hormone statuses and found the heights and SDS for heights in hypothyroidism group, hypoadrenalism group and hypogonadism group were higher than that in their control group. That indicated pituitary multiple hormone deficiencies other than growth hormone deficiency may contribute to the higher height in PSIS than IGHD. For instance, E2 and T are very important for the epiphyseal closure. Deficiency of these two hormones may result in the delay of bone age and difficulty in the closure of epiphysis. This could be supported further by our correlation analysis that multiple hormone correlated with height and SDS height and by the much larger differences between actual age and bone ages in PSIS than that in IGHD.

Gonadotrophin deficiency is a common finding in patients with PSIS. However, its severity is variable, ranging from complete gonadotrophin deficiency to normogonadotropic amenorrhoea. We found that 9.1% of patients in this group presented for lack of puberty or poor development of secondary sex characteristics, and some patients have been diagnosed with idiopathic hypogonadotrophic hypogonadism. As a matter of fact, in our further investigation, all males with PSIS had absent pubertal development and only 2 female patients had partial pubertal development, and 95.8% of this group of PSIS patients over 14 years (boys) or 13 years (girls) exhibited poor secondary sexual characteristic development due to gonadotrophin deficiency, which was much higher than that reported in literature [27], in which, out of 27 patients, five displayed spontaneous full pubertal development with normal hormonal values and 22 of 27 patients (81%) had pubertal deficiency.

The frequency of multiple anterior pituitary deficiencies in PSIS varied in different series. In our series, we found 51/55 (92.7%) had three or more kinds of anterior pituitary dysfunction, which was much higher compared with reports in the literature. For example, Arrigo et al. [24] reported a frequency of 64.7% of multiple anterior pituitary deficiencies in 17 PSIS patients. Another analysis on 83 cases of PSIS revealed the incidence of deficiency of multiple hormones from anterior pituitary was up to 87.5% [7]. The same trends were found by Marcu et al. [28] and Rottembourg et al. [27], though Coutant et al. [25] reported 15/15 cases of multiple anterior pituitary deficiencies compared with one in 48 patients with non-acquired GH deficiency with normal pituitary MRI. So, the incidence rates of combined hormones deficiency of various anterior pituitary hormones differed significantly in different reports. In our study, the prevalence of deficiencies in growth hormone, gonadotropin, corticotrophin and thyrotropin were 100% (100% complete GHD), 95.8%, 81.8% and 76.3% respectively, while in the series of 35 PSIS patients from Tauber et al. [8] the prevalences were 100% (80% with complete GHD and 20% partial GHD), 47.1%, 33.3% and 41.4%, respectively. In the publications that provide details of deficiencies, thyrotropin deficiency appears to be the most frequent, ranging from 70.3% to 91% [8], [20], [27], [29]. Analysis of gonadotropin deficiency depends on the pubertal status of patients at the time of the evaluation. In patients diagnosed in infancy and then re-evaluated at the end of their growth, or at a stage of physiological pubertal development, the frequency of gonadotropin deficiency varied between 43% and 86% [8], [27]–[29]. This finding underlines the importance of periodic follow-up of pituitary hormone status as well as sexual development in these children, at least until they have reached their adult height.

Pituitary stalk lesions, which can interrupt normal dopaminergic inhibition of PRL, often result in hyperprolactinemia. Our study found the occurrence of hyperprolactinemia in PSIS was 36.4% and hypoprolactinemia was absent. While in a series of 27 PSIS subjects [27], one patient had PRL deficiency and only two patients had hyperprolactinemia. One reasonable explanation for this big difference may be the heterogeneous degree of the dopaminergic pathway disconnection.

Anterior pituitary and posterior pituitary have different physiogenesis and blood supply [30]. Patients with PSIS have a disorder of connecting between pituitary and hypothalamus, which leads to the hypogenesis of anterior pituitary and the posterior pituitary not entering the hypophysial fossa. Clinically, insufficiency of posterior pituitary among patients with PSIS was rare. Like Tauber, et al. [8], Marcu, et al. [28] and Chen, et al. [20], we found no case of diabetes insipidus. However in Fernandez-Rodriguez, et.al., 8.3% of PSIS patients had the symptoms of polydipsia and polyuria [6], but the diagnosis of diabetes insipidus remained to be confirmed.

In our study, PSIS was diagnosed primarily by the help of MRI, and its characteristic imaging was dysplasia of anterior pituitary, absence of pituitary stalk and ectopic posterior pituitary which was located near the optic chiasm and hypothalamus. All patients in this group had no visible pituitary stalk; no patient with thin pituitary stalk was found. The average height of anterior pituitary was 28 mm, which was similar to the report of others [31]. MRI plays an important role in the diagnosis of PSIS among patients initially presented with growth failure.

In the literature [8], [13], about 20–50% of the PSIS patients also had developmental malformations of structures or organs along the midline of central nervous system, e.g., cleft lip, diaphragm absence, hypoplasia of optic nerve, bulging brain, harelip or other deformity, which indicated that the gene defect corresponding to this disease may be related to the gene charging the embryonic development of hypothalamus-pituitary area. However, in our study midline abnormalities were present in 9.1% of PSIS patients. Development defect of structures along the midline of central nervous system give rise to an extra support to explore some genetic etiology for PSIS.

In general, the clinical features of our Chinese PSIS patients seem to be different from other reported patients in regarding to the higher degree of hypopituitarism and lower prevalence of midline defects. In addition, our patients were older at the time of case detection and the bone age was markedly delayed. We also had no cases of familial PSIS.

## Methods

### 1. Subjects

From Jan. 2000 to Oct. 2011, we collected 55 cases of PSIS diagnosed among the 251 hospitalized hypopituitarism patients in our hospital, including 48 males and 7 females. The geographic distribution of patients included 17 from Northern China, 13 from Southern China, 11 from Eastern China and 14 from Western China. The diagnosis had been confirmed by MRI examination, dynamic endocrine evaluation of anterior pituitary function, and clinical features. The perinatal characteristics of these patients (measurements, term and conditions of birth, associated malformations) and the positions of fetus at birth and whether or not anoxia occurred were recorded. No patients had relevant family history. This study has been approved by Ethics Committee of Chinese PLA General Hospital and due to the retrospective nature of the study, informed consent was waived. The subject of the photograph gave written informed consent, as outlined in the PLOS consent form, to publication of her photograph.

### 2. Clinical Data and Methods

#### 2.1 The medical records were retrospectively reviewed

Consanguineous parents, family history, perinatal histories (including gestational age, delivery and neonatal hypoxemia), associated malformations (including microphallus, cryptorchidism and midline abnormalities), pubertal status, chronological age, bone age, height and so on were recorded. Height was expressed in standard deviation scores (SDS) for chronological age (CA). Short stature was classically defined by height less than −2 SDS for the CA. Microphallus was defined as a penis length less than or equal to 2.5 cm [13].

#### 2.2 Comprehensive evaluation of anterior pituitary function

(a) Secretion of growth hormone: Thesecretory status of growth hormone (GH) was evaluated by pyridostigmine bromide test (2 mg/kg, 60 mg/tab, Sunway Pharmaceutical Co., Ltd.,Shanghai) and by insulin-induced hypoglycemia tolerance test (ITT) (0.1 IU/kg, Regular insulin, Novo Nordisk, Denmark). Complete GH deficiency and partial GH deficiency were confirmed when the peak GH values in both tests were less than 10 ng/ml and 5 ng/ml respectively [14]. (b) Gonadotroph axis: LH, FSH, testosterone, and estradiol levels were tested, and the gonadotropin-releasing hormone (Gonadorelin 100 ug, Maanshan Fengyuan Pharmaceutical Co., Ltd., China) stimulating test was performed; hypogonadism was diagnosed on the basis of delayed or absent pubertal development with low serum testosterone (below 8.4 nmol/L for male) (normal range: 8.4–28.7 nmol/L) or estradiol (below 48.2 pmol/L for female) (normal range: 48.2–905.4 pmol/L) levels and blunted LH/FSH response to a Gonadorelin stimulation test. Prolactin (PRL) deficiency and hyperprolactinemia were defined as basal serum PRL lower than 2.1 ug/L for male (normal range: 2.1–17.7 ug/L), 2.8 ug/L for female (normal range: 2.8–29.2 ug/L) and higher than 17.7 ug/L for male, 29.2 ug/L for female respectively. (c) Hypothalamic-pituitary-adrenal (HPA) axis: The cortisol and corticotropin (ACTH) levels at 8 am and the 24-hour urinary free cortisol exretion were determined, and the ACTH response was determined with the ITT; HPA axis hypofunction was idenfied when the basal serum cortisol concentration (normal range: 198.7–797.5 nmol/L) was below 198.7 nmol/L or the ratio of ACTH peak value to the basal ACTH value after ITT was less than 3. (d) Pituitary-thyroid function: The levels of thyroid-stimulating hormone (TSH), T_4_, T_3_, free T_4_ (FT_4_), and free FT3 were determined. The diagnosis of hypothyroidism was made if the basal serum FT4 (normal range: 10.4–24.3 pmol/L) was equal to or lower than 10.4 pmol/L.

#### 2.3 Assays and quantification

All the hormones in the study were analyzed by chemiluminescence immunoassay. ACTH and GH were detected using an Immulite 2000 Analyzer (Siemens Healthcare Diagnostics Inc, Los Angeles, USA), while all others were measured with an ADVIA Centaur Analyzer (Siemens Healthcare Diagnostics, Tarrytown, NY, USA). The differences between and within-batch were 2.22–4.69% and 1.58–4.59% for free thyroxine, 2.89–3.69% and 1.86–5.45% for 8 am cortisol, 2.3–6.2% and 1.4–4.7% for testosterone in males, 4.0–12.1% and 4.5–8.1% for estrogen in females, and 2.3–3.3% and 1.4–4.7% for prolactin, respectively. All blood samples were obtained in the morning and in the fasted state.

#### 2.4 Imaging evaluation

Magnetic resonance imaging (MRI) (Signa HDxt 1.5T, GE Company, USA) included dedicated sellar images with coronal and sagittal imaging after injection of Dimeglumine Gadopentetate. Bone age (BA) was determined using an X-ray image of the left hand and wrist according to Greulich and Pyle method.

### 3. Statistical Analysis

The data were analyzed based on the presence or absence of hypogonadism, hypoadrenalism, hypothyroidism or hyperprolactinemia. Pituitary-target gland functional status was also analyzed based on age (year), sex ratio, breech prevalence, height, -SDS height, ratio of upper to lower body segment (RUL), ratio of arm span to height (RAH), bone age, basal GH, peak GH, TSH, FT_4_, basal ACTH, peak ACTH, 8 am serum cortisol (F 8 am), testosterone (T), estradiol (E_2_), basal Luteinizing hormone (LH), peak LH, and prolactin (PRL). In addition, we analyzed the correlation between height or –SDS height and hormone status. Data were expressed as means ± standard deviations, percentage or median (interquartile range). Univariate comparisons between groups were performed by t-test for variables with normally distributed or Mann-Whitney U test and χ2 test when necessary. Correlations between height or SDS height and hormone secretion level were performed by Pearson correlation or Spearman correlation if necessary. *P*<0.05 was considered statistically significant. Statistical tests were performed using the SPSS17.0 statistical package.
